# Comparative Outcomes of Two Non‐Crosslinked Porcine Acellular Dermal Matrices in Complex Abdominal Wall Reconstruction: A Randomized Controlled Trial and an Observational Cohort Study

**DOI:** 10.1002/wjs.70271

**Published:** 2026-02-20

**Authors:** Abdulaziz Elemosho, Benjamin A. Sarac, Molly A. Olson, Ibrahim Khansa, Paige N. Hackenberger, Vimal Narula, Daniel S. Eiferman, Jeffrey E. Janis

**Affiliations:** ^1^ Department of Plastic and Reconstructive Surgery, College of Medicine The Ohio State University Wexner Medical Center Columbus Ohio USA; ^2^ Independent Researcher; ^3^ Plastic and Reconstructive Surgery Texas Children's Hospital‐North Austin Austin Texas USA; ^4^ Baylor College of Medicine Houston Texas USA; ^5^ Division of Plastic and Reconstructive Surgery, Department of Surgery Northwestern University Feinberg School of Medicine Chicago Illinois USA; ^6^ Department of Surgery The Ohio State University Wexner Medical Center Columbus Ohio USA

**Keywords:** abdominal wall reconstruction, biologic mesh, biologics, non‐crosslinked, porcine acellular dermal matrix, strattice, surgical site occurrences, ventral hernia repair, XenMatrix, xenograft

## Abstract

**Background:**

Biologic mesh has historically been used for ventral hernia repairs (VHR) in contaminated fields in an off‐label fashion due to early evidence suggesting that they may be able to withstand these conditions more favorably than synthetic mesh. This study aims to compare outcomes of two non‐crosslinked porcine acellular dermal matrices—XenMatrix (Bard, Covington, GA) and Strattice (LifeCell Corporation, Bridgewater, NJ) used in VHR.

**Methods:**

Patients who were undergoing elective open VHR were randomized to receive either XenMatrix or Strattice mesh (randomized controlled trial—RCT cohort). An additional cohort of patients were recruited in a retrospective observational study cohort. Surgical site occurrence (SSO) was the primary outcome evaluated with hernia recurrence being a secondary outcome measure. Simple and multivariate logistic regression analyses were conducted separately for the RCT and observational cohorts.

**Results:**

Forty‐six patients were randomized into the RCT cohort, and an additional 20 patients were recruited into the observational study cohort. There was no difference in baseline characteristics between the two mesh groups in both the RCT and observational cohorts. In the RCT cohort, the 6‐week SSO rate was significantly higher for XenMatrix (36.7%) than Strattice (6.3%) (*p* = 0.03), and on multivariate analysis, XenMatrix was associated with higher 6‐week SSO risk than Strattice [OR: 19.5 (95% CI: 2.3–523.7) and *p* = 0.02]. However, in the observational cohort, the rate of 6‐week SSO was similar for both XenMatrix (50.0%) and Strattice (33.3%) (*p* = 0.46) as well in the multivariate analysis [OR: 6.6 (95% CI: 0.4–324.6) and *p* = 0.23]. Finally, random effect meta‐analysis of 6‐week risk of SSO of both RCT and observational cohort showed that XenMatrix is associated with higher 6‐week SSO risk than Strattice [OR: 12.5 (95% CI: 1.8–89.2) and *I* ^2^ = 0% *p* = 0.012].

**Conclusion:**

Our study showed that XenMatrix may be associated with higher risk of early SSO compared to Strattice. This underscores the importance of more head‐to‐head mesh comparison to optimize outcomes following VHR.

**Trial Registration:**

NCT02228889 (www.clinicaltrials.gov)

## Graphical abstract

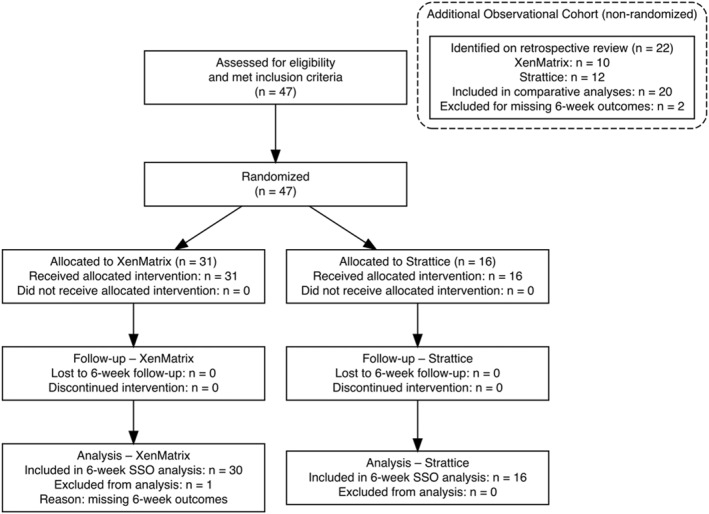

## 1 Introduction

More than 250,000 ventral hernia repairs are performed in the United States every year [1, 2], with surgical site occurrences (SSOs) as well as hernia recurrences remaining a significant problem [3, 4]. When a patient requires a reoperation for any complication following initial hernia repair, the risk of subsequent reoperation increases significantly [5]. In fact, Flum et al. have shown that the 5‐year risk of reoperation increases stepwise with every reoperation [5] (24% after the first reoperation, 35% after the second reoperation, and 39% after the third reoperation). This underscores the importance of performing the best possible hernia repair the first time. Several studies have reported that mesh‐reinforced ventral hernia repairs are less likely to recur than repairs without mesh [6, 7]. Bhardwaj et al. recently found 5‐year hernia recurrence risk to be 40% with mesh repair and 70% following repair without mesh [6].

A critical contributing factor in the success of hernia repair that is still debated is the ideal type of mesh [8, 9]. The type of mesh used in hernia repair falls into three categories: synthetic, biologic, and bioresorbable. Synthetic meshes reinforce the strength of the fascial repair but have been historically associated with a high rate of infection and enterocutaneous fistula formation, especially heavyweight microporous synthetic meshes placed in contaminated fields and/or in direct contact with the viscera [2, 10, 11]. Biologic mesh consists of acellular collagen matrices from different biological sources (human, porcine, bovine, and equine), that are less prone to fibrous encapsulation than synthetic meshes as long as they are non‐crosslinked [12, 13, 14]. There is additional evidence that biologic mesh can also serve as scaffold that allows ingrowth of new tissue, including new blood vessels, which was originally thought to make them less prone to infection than synthetic mesh [11]. Finally, long‐acting bioresorbable meshes combine the structural strength of synthetic meshes with the remodeling advantages of biologic materials. Recent evidence suggests they may offer the most favorable long‐term outcomes [15]; however, their cost remains a major limitation to widespread use [15, 16, 17].

Historically, it was thought that biologic mesh was superior to synthetic mesh in certain clinical situations due to these previously mentioned characteristics [18]. The Ventral Hernia Working Group (VHWG) even suggested that using a biologic mesh may be more advantageous than a synthetic mesh for patients with a VHWG hernia grade of two or higher [2]. However, recent studies have found synthetic mesh and bioresorbable mesh to have comparable or better outcomes than biologic mesh even in contaminated fields [15, 19, 20, 21].

Nevertheless, given the widespread use of biologic mesh in contaminated fields (off‐label), and the paucity of head‐to‐head analyses of the different biologic meshes, our study sought to compare outcomes of two different non‐crosslinked porcine acellular dermal matrices (ADM), specifically XenMatrix (Bard, Covington, GA) and Strattice (LifeCell Corporation, Bridgewater, NJ).

## 2 Methods

### 2.1 Study Design

A randomized controlled trial (RCT) designed for patients undergoing open elective complex abdominal wall reconstruction with planned biologic mesh between February 2015 and August 2022. This RCT is reported in accordance with the CONSORT 2010 guidelines with the completed checklist provided as Supporting Information S1. Subjects were recruited if they fulfilled all inclusion criteria.

### 2.2 Participants and Study Settings

All surgical candidates were optimized according to a previously described protocol [22]. All participants provided voluntary informed consent prior to enrollment, in accordance with IRB‐approved study protocols. Eligible patients were adults over 18 years old, suitable for elective hernia repair with VHWG grade 2 or above. Patients who did not provide consent were excluded from the study. Complete inclusion or exclusion criteria can be found in the Supporting Information S1. Patient recruitment and surgical procedures were performed at the Ohio State University Wexner Medical Center.

The trial was registered on clinicaltrial.gov, (NCT02228889) after ethical approval was acquired from the Institutional Review Board (IRB) of the Ohio State University.

### 2.3 Surgical Intervention

All procedures were performed by the senior author (J.E.J.) together with one of the coauthor general surgeons (either D.S.E. or V.N.), with special expertise in complex abdominal wall reconstruction. Patients were managed using a standardized perioperative protocol including preoperative optimization, planned use of component separation when indicated, and preference for primary fascial closure with mesh reinforcement when feasible. Closed‐suction drainage was used in all patients.

### 2.4 Sample Size, Randomization, and Blinding

A power analysis was performed based on outcomes from two industry‐sponsored studies for XenMatrix mesh [11, 12], and another study by Clemens et al. for Strattice mesh [23]. Assuming an alpha error of 5%, a power of 80%, and assuming a large standard deviation (3), the minimum total number of patients that was needed to demonstrate a significant difference in our primary outcome was determined to be 70 patients (35 in each arm).

Eligible patients who provided informed consent were randomized in a 1:1 fashion to receive either Strattice or XenMatrix as the biologic mesh for abdominal wall reconstruction. The allocation sequence was generated a priori using a computerized random number generator in Microsoft Excel (group A = Strattice, group B = XenMatrix) by a study team member who was not involved in patient recruitment or intraoperative decision‐making. Simple randomization without blocking or stratification was used. Randomization was implemented using sequentially numbered, sealed, and opaque envelopes that were opened only after the patient's eligibility and willingness to participate had been confirmed. The trial was conducted as a single‐blinded study: patients were not informed which mesh they received, whereas the operating surgeon and operating room staff necessarily knew the assigned product for procedural and documentation purposes. No formal interim analyses or statistical stopping rules were planned. The decision to terminate enrollment was driven by declining accrual and institutional formulary changes as detailed below. Postoperative clinical assessments and data abstraction were performed by senior surgeon (J.E.J) and research staff who were not blinded to the assigned mesh, whereas patients remained blinded throughout follow‐up. The two meshes were handled and implanted using the same operative principles and perioperative pathways, which helped maintain patient blinding.

### 2.5 Outcome Measures

The primary outcome of this study was surgical site occurrence (SSO) at 6 weeks as the majority of SSOs typically present within the early postoperative period. Hernia recurrence was evaluated as a secondary outcome, with all cohorts having a minimum follow‐up of 1 year. Surgical site occurrences (SSOs) were defined as any occurrence of skin necrosis, fat necrosis, wound dehiscence, infection, hematoma, seroma, and enterocutaneous fistula [24]. Seroma and hematoma were defined as confirmed collections of serous fluid and blood, respectively, requiring drainage [25]. Infection refers to any infectious processes, such as cellulitis and abscess formation, that required intravenous or oral antibiotic use with or without drainage procedures [25]. Hernia recurrence is defined as an abnormality in the abdominal wall that included a fascial defect [25]. No changes were made to the prespecified primary (6‐week SSO) or secondary (hernia recurrence) outcomes after trial commencement.

### 2.6 Data Collection

Hernia recurrence was initially detected through history and physical examination and was subsequently objectively confirmed with abdominal imaging. Clinical evaluation was documented at a structured follow‐up schedule (weekly for 1 month, then every 3 months up to 1 year) for assessment of SSOs, recurrence, and other complications following the standard definition above. All SSOs were confirmed clinically. The datapoints collected are presented in the Supporting Information S1.

### 2.7 Termination of Trial

During recruitment and enrollment in the study, the number of patients enrolled declined over time. This was due to additional published evidence that favored synthetic mesh over biologic mesh as well as system financial issues with maintaining more expensive biologic mesh on formulary. Similar trends were seen around the United States and world as growing evidence showed support for synthetic mesh, even in the setting of contaminated hernias [26]. A total of 46 patients were enrolled in the original RCT, which was insufficient from a power analysis standpoint.

To increase sample size, a comprehensive retrospective chart review of the electronic health records (EHRs) was conducted to identify all patients who met our initial RCT criteria following the IRB approval of waiver of informed consent. The two resultant datasets were synthesized to generate an overall estimate of the treatment effect of mesh type on our primary outcome. This approach has been described and utilized by several studies [27, 28, 29, 30, 31].

### 2.8 Statistical Analysis

#### 2.8.1 Baseline Characteristics

Descriptive statistics (e.g., mean or median for continuous data, counts, and proportions for categorical data) were used to summarize baseline characteristics of each the RCT and observational cohorts. In both RCT and observational cohorts, continuous variables were compared using the Kruskal–Wallis test and categorical variables were compared using Fisher’s exact test.

#### 2.8.2 Primary Outcome Analysis

SSO within 6 weeks was analyzed using logistic regression. Both simple and multivariable logistic regression models were fit for each RCT and for the observational cohort, with mesh type (XenMatrix vs. Strattice) as the primary predictor. Multivariable models adjusted for hernia width, CDC wound class, immunosuppression, and recurrent hernia to account for potential confounding. Potential confounders were reviewed and selected prior to model fitting based on expert opinion.

After estimating effects separately for the RCT and observational cohort, a random‐effects meta‐analysis was performed to generate a combined estimate [27, 30, 31]. The pooled log odds ratio was calculated using inverse variance weighting and a restricted maximum likelihood estimator. Between‐study heterogeneity was assessed by the *I* ^2^ measure.

Forest plots and summary tables for the log odds ratio and odds ratio were generated to summarize results. All statistical analysis was performed using R Studio (4.4.1) and statistical significance was set at *p* < 0.05.

## 3 Results

**FIGURE 1:**
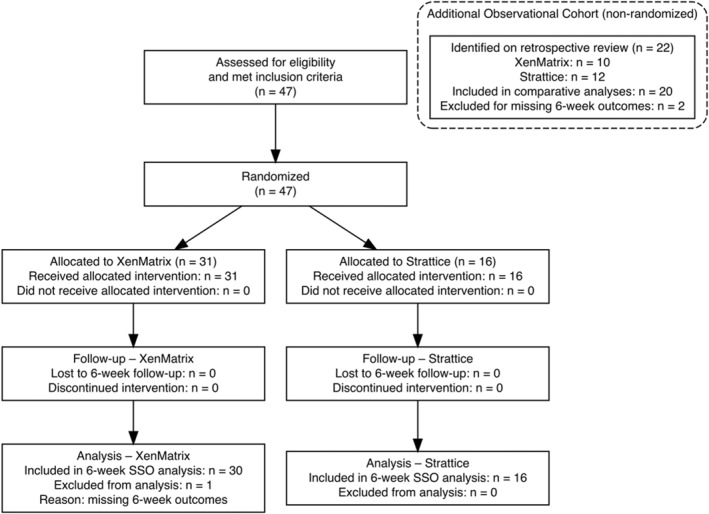
CONSORT flow diagram.

A total of 47 patients were screened for eligibility and met inclusion for the randomized trial. All patients provided consent and were randomized (XenMatrix *n* = 31 and Strattice *n* = 16) to receive the allocated mesh. In addition, 22 observational patients (XenMatrix *n* = 10 and Strattice *n* = 12) met the same eligibility criteria on retrospective review. No patients were excluded from the analyses for protocol violations; however, three patients from XenMatrix group were excluded (one from the randomized trial and two from the observational cohort) due to an incomplete 6‐week outcome data Figure 1.

### 3.1 RCT Cohort

#### 3.1.1 Patient Demographics and Baseline Characteristics

Thirty (65.2%) patients had AWR with XenMatrix, and 16 (34.8%) patients had AWR with Strattice. Mean follow‐up period for Strattice was 1191.4 ± 774.6 days (Range: 76.0–3100.0 days) versus XenMatrix 710.1 ± 632.6 days (Range: 0.0–2157.0 days) *p* = 0.02.

There were no significant differences in body mass index [32.2 ± 6.2 kg/m^2^ vs. 30.1 ± 5.0 kg/m^2^ (*P* = 0.27)] and mean defect size (195.6 ± 157.5 cm^2^ vs. 266.0 ± 200.9 cm^2^ *P* = 0.26) between the XenMatrix versus Strattice groups, respectively. Table 1 shows other baseline comorbidities within the two groups.

**TABLE 1:** Baseline characteristics—RCT.

Variables	Strattice (*N* = 16)	XenMatrix (*N* = 30)	*p* value
Sex: Male	6 (37.5%)	16 (53.3%)	0.31
Days from surgery to last follow‐up (mean ± SD)	1191.4 ± 774.6	710.1 ± 632.6	0.02
Age (mean ± SD)	59.3 ± 13.8	53.6 ± 13.5	0.12
BMI (mean ± SD)	30.1 ± 5.0	32.2 ± 6.2	0.27
BMI > 30 kg/m^2^	8 (50.0%)	18 (60.0%)	0.51
Diabetes	7 (43.8%)	7 (23.3%)	0.15
Hypertension	7 (43.8%)	14 (46.7%)	0.85
COPD	5 (31.3%)	5 (16.7%)	0.25
Immunosuppression	2 (12.5%)	10 (33.3%)	0.13
Primary hernia repair	4 (25.0%)	12 (40.0%)	0.50
Parastomal hernia	2 (12.5%)	1 (3.3%)	
Recurrent hernia repair	12 (75.0%)	18 (60.0%)	0.89
Parastomal hernia	1 (6.3%)	1 (3.3%)	
Enterocutaneous fistula	1 (6.3%)	0	
Number of prior hernia repairs (median, IQR)	2.50 (0.0–4.6)	1.00 (0.0–2.0)	0.17
History of mesh repair	12 (75.0%)	17 (56.7%)	0.22
Hernia area (mean ± SD)	266.0 ± 200.9	195.6 ± 157.5	0.26
Total OR time (mean ± SD)	462.6 ± 58.2	451.2 ± 142.8	0.59
Closed incisional negative‐pressure wound therapy	11 (68.8%)	8 (26.7%)	0.01
ASA class			0.33
2	3	3	
3	13	24	
4	0	3	

#### 3.1.2 Surgical Characteristics

Groups did not differ in the type of hernia diagnosis, CDC wound class, or Ventral Hernia Working Group VHWG classification (Table 2). There was no difference in the technique used for defect closure between the XenMatrix versus Strattice groups: Primary fascial closure [73.3% vs. 87.5% (*P* = 0.27)] versus bridged interposition mesh repair [20.0% vs. 12.5% (*P* = 0.52)]. Similarly, there was no difference in the mesh position placement between the XenMatrix versus Strattice groups: onlay [10.0% vs. 0% (*P* = 0.19)], retrorectus [26.7% vs. 25.0% (*P* = 0.90)], bridging inlay [6.7% vs. 0% (*P* = 0.29)], and wide intraperitoneal underlay [80.0% vs. 75.0% (*P* = 0.70)] (Table 2).

**TABLE 2:** Surgical characteristics—RCT.

Variables	Strattice (*N* = 16)	XenMatrix (*N* = 30)	*p* value
Ventral hernia	15 (93.8%)	28 (93.3%)	0.96
CDC wound class			0.38
1	7	17	
2	5	4	
3	2	2	
4	2	7	
VHWG grade			0.33
1	1	1	
2	2	9	
3	11	13	
4	2	7	
Kanters grade			0.57
1	1	1	
2	6	16	
3	9	13	
Primary fascial repair	14 (87.5%)	22 (73.3%)	0.27
Unilateral component separation	2 (12.5%)	2 (6.7%)	0.50
Bilateral component separation	8 (50.0%)	17 (56.7%)	0.67
Bridged repair	2 (12.5%)	6 (20.0%)	0.52
Onlay mesh placement	0 (0.0%)	3 (10.0%)	0.19
Underlay mesh placement	12 (75.0%)	24 (80.0%)	0.70
Inlay mesh placement	0 (0.0%)	2 (6.7%)	0.29
Sublay mesh placement	4 (25.0%)	8 (26.7%)	0.90

#### 3.1.3 Surgical Outcomes

At 6‐week postoperative follow‐up, the XenMatrix group had a higher rate of SSO than the Strattice group in the randomized cohort [36.7% vs. 6.3% and *p* = 0.03]. The adjusted 6‐week SSO risk was higher in the XenMatrix group than in the Strattice group in the randomized cohort [OR: 19.5 (95% CI: 2.3–523.7) and *p* = 0.02]. There was no recurrence or bulge seen in either arm of the randomized cohort at the mean follow‐up period. Finally, XenMatrix similarly did not have statistically different rates of reintervention (23.3% vs. 12.5% and *p* = 0.46), and there was no evidence that readmission (33.3% vs. 18.8% and *p* = 0.49) was different from Strattice, respectively (Table 3).

**TABLE 3:** 6‐Week outcomes—RCT.

Variables	Strattice (*N* = 16)	XenMatrix (*N* = 30)	*p* value
Bulge	0 (0.0%)	0 (0.0%)	—
Recurrence	0 (0.0%)	0 (0.0%)	—
SSO within 6 weeks	1 (6.3%)	11 (36.7%)	0.03
Underlay mesh	1/12 (8.3%)	7/17 (41.2%)	0.09
Sublay mesh	0/4 (0%)	1/6 (16.7%)	1.00
Onlay mesh	0	3/7 (42.9%)	—
Closed incision wound Vac	1/11 (9.1%)	4/8 (50.0%)	0.11
Skin necrosis	0 (0.0%)	1 (3.3%)	0.46
Enterocutaneous fistula	0 (0.0%)	2 (6.7%)	0.29
Dehiscence	0 (0.0%)	1 (3.3%)	0.46
Seroma	0 (0.0%)	2 (6.7%)	0.29
Hematoma	0 (0.00%)	0 (0.0%)	—
Infection	1 (6.3%)	10 (33.3%)	0.04
Mesh infection	0 (0.0%)	3 (10.0%)	0.19
Reintervention within 6 weeks	2 (12.5%)	7 (23.3%)	0.46
Readmission within 6 weeks	3 (18.8%)	10 (33.3%)	0.49

### 3.2 Observational Cohort

#### 3.2.1 Patient Demographics and Baseline Characteristics

Eight (40.0%) patients had AWR with XenMatrix, and 12 (60.0%) patients had AWR with Strattice. Follow‐up duration was not statistically different between the XenMatrix (715.4 ± 669.5 days S.D and range: 64.0–1751.0 days) and Strattice groups (327.1 ± 266.9 days S.D and range: 54.0–855.0 days) *p* = 0.29.

There were no significant differences in body mass index [31.2 ± 6.5 kg/m^2^ vs. 35.6 ± 6.0 kg/m^2^ (*P* = 0.15)] and mean defect size (398.5 ± 130.5 cm^2^ vs. 298.8 ± 189.0 cm^2^ *P* = 0.12) between the XenMatrix versus Strattice groups, respectively. Table 4 shows other baseline comorbidities within the two groups.

**TABLE 4:** Baseline characteristics (observational patients).

Variables	Strattice (*N* = 12)	XenMatrix (*N* = 8)	*p* value
Sex: Male	6 (50.0%)	3 (37.5%)	0.58
Days from surgery to last follow‐up (mean ± SD)	327.1 ± 266.9	715.4 ± 669.5	0.29
Age (mean ± SD)	54.8 ± 10.2	53.12 ± 17.0	0.94
BMI (mean ± SD)	35.6 ± 6.0	31.2 ± 6.5	0.15
BMI > 30 kg/m^2^	10 (83.3%)	5 (62.5%)	0.29
Diabetes	2 (16.7%)	0 (0.0%)	0.22
Hypertension	7 (58.3%)	2 (25.0%)	0.14
COPD	1 (8.3%)	1 (12.5%)	0.76
Immunosuppression	4 (33.3%)	0 (0.0%)	0.07
Primary hernia repair	4 (33.3%)	5 (62.5%)	0.50
Parastomal hernia	2 (16.7%)	0	
Recurrent hernia repair	8 (66.7%)	3 (37.5%)	0.89
Parastomal hernia	2 (16.7%)	0	
Enterocutaneous fistula	2 (16.7%)	0	
Number of prior hernia repairs (median, IQR)	1.00 (0.0–1.6)	0.00 (0.0–2.2)	0.57
History of mesh repair	6 (50.0%)	3 (37.5%)	0.58
Hernia area (mean ± SD)	284.3 (231.3–422.2)	362.9 (322.1–467.2)	0.12
Total OR time (mean ± SD)	431.00 (383.2–540.6)	551.00 (345.2–575.8)	0.57
Closed incisional negative‐pressure wound therapy	11 (91.7%)	6 (75.0%)	0.54
ASA class			0.17
2	1	1	
3	11	5	
4	0	2	

#### 3.2.2 Surgical Characteristics

Groups did not differ in the type of hernia diagnosis, CDC wound class, or Ventral Hernia Working Group VHWG classification (Table 5). There was no difference in the technique used for defect closure between the XenMatrix versus Strattice groups: Primary fascial closure [85.7% vs. 75.0% (*P* = 0.58)] and bridged interposition mesh repair [14.3% vs. 33.3% (*P* = 0.36)]. Between the XenMatrix versus Strattice groups, there was no difference in the mesh position placement (retrorectus [25.0% vs. 0% (*P* = 0.07)], bridging inlay [12.5% vs. 8.3% (*P* = 0.76)], and wide intraperitoneal underlay [75.0% vs. 100.0% (*P* = 0.07)]) (Table 5).

**TABLE 5:** Surgical characteristics—observational patients.

Variables	Strattice (*N* = 12)	XenMatrix (*N* = 8)	*p* value
Ventral hernia	11 (91.7%)	7 (87.5%)	0.76
CDC wound class			0.30
1	6	5	
2	3	2	
3	0	1	
4	3	0	
VHWG grade			0.21
1	0	1	
2	6	2	
3	4	5	
4	2	0	
Kanters grade			0.36
1	0	0	
2	5	5	
3	7	3	
Primary fascial repair	9 (75.0%)	6 (85.7%)	0.58
Unilateral component separation	1 (8.3%)	0 (0.0%)	0.40
Bilateral component separation	4 (33.3%)	7 (87.5%)	0.02
Bridged repair	4 (33.3%)	1 (14.3%)	0.36
Onlay mesh placement	0 (0.0%)	0 (0.0%)	—
Underlay mesh placement	12 (100.0%)	6 (75.0%)	0.07
Inlay mesh placement	1 (8.3%)	1 (12.5%)	0.76
Sublay mesh placement	0 (0.0%)	2 (25.0%)	0.07

#### 3.2.3 Surgical Outcomes

There were no statistically significant differences in 6‐week SSO rates between the XenMatrix and Strattice groups [50.0% and 33.3% and *p* = 0.46, respectively]. Similarly, the adjusted 6‐week SSO risk was not different between the mesh groups [OR: 6.6 (95% CI: 0.4–324.6) and *p* = 0.23]. The recurrence rate was not statistically different in the XenMatrix (0%) and Strattice (8.3%) groups [*p* = 0.40] at their mean follow‐up time. Finally, XenMatrix similarly did not have statistically different rates of reintervention (37.5% vs. 16.7% and *p* = 0.347), and readmission (50.0% vs. 16.7% and *p* = 0.161) was not different than Strattice, respectively Table 6.

**TABLE 6:** 6‐Week outcomes—observational patients.

Variables	Strattice (*N* = 12)	XenMatrix (*N* = 8)	*p* value
Bulge	0 (0.0%)	0 (0.0%)	—
Recurrence	1 (8.3%)	0 (0.0%)	0.40
SSO within 6 weeks	4 (33.3%)	4 (50.0%)	0.46
Underlay mesh	4/11 (36.4%)	3/5 (60.0%)	0.60
Sublay mesh	—	1/2 (50.0%)	—
Closed incision wound Vac	3/11 (27.3%)	2/6 (33.3%)	1.00
Skin necrosis	0 (0.0%)	1 (12.5%)	0.21
Enterocutaneous fistula	2 (16.7%)	0 (0.0%)	0.22
Dehiscence	2 (16.7%)	0 (0.0%)	0.22
Seroma	1 (8.3%)	0 (0.0%)	0.40
Infection	1 (8.3%)	3 (37.5%)	0.11
Mesh infection	0 (0.0%)	0 (0.0%)	—
Reintervention within 6 weeks	2 (16.7%)	3 (37.5%)	0.35
Readmission within 6 weeks	2 (16.7%)	4 (50.0%)	0.16

### 3.3 Meta‐Analysis

**FIGURE 2:**
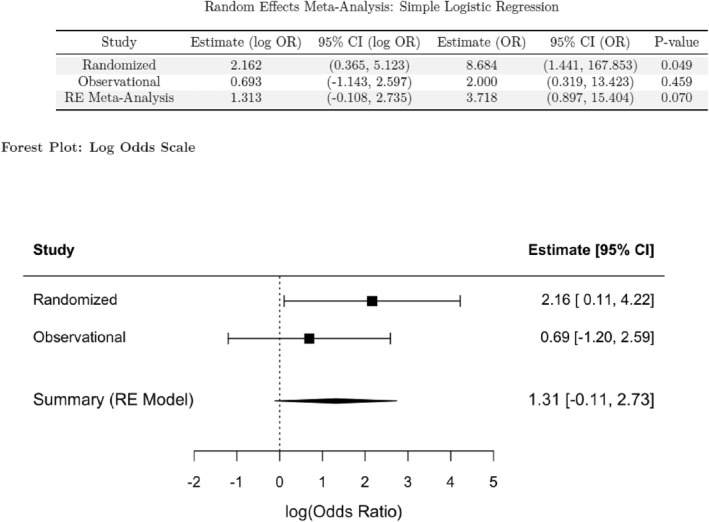
Unadjusted meta‐analysis of 6‐week SSO risk.

**FIGURE 3:**
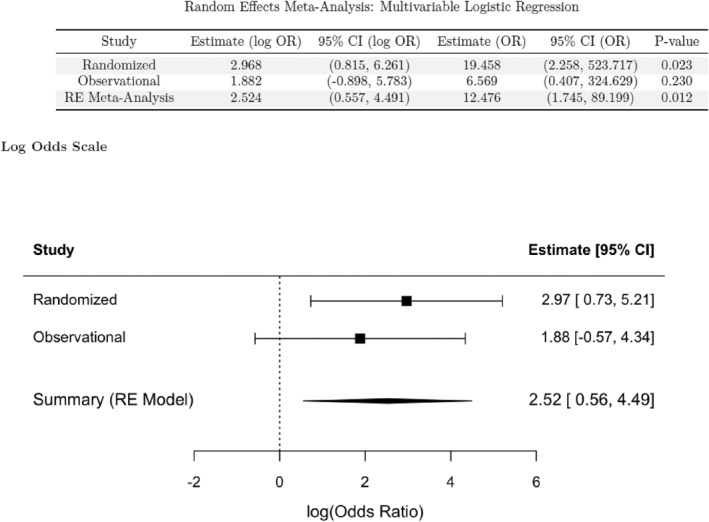
Adjusted meta‐analysis of 6‐week SSO risk.

The overall rate of 6‐week SSO for both PADMs was 26.1% in the randomized cohort and 40.0% in the observational cohort. Random‐effect meta‐analysis showed no significant difference in unadjusted SSO risk between the XenMatrix and Strattice groups [OR: 3.7 (95% CI: 0.9–15.4) and *I* ^2^ = 3.3% *p* = 0.07] (Figure 2), but showed a higher SSO risk in the XenMatrix group than in the Strattice group after covariate adjustment [OR: 12.5 (95% CI: 1.8–89.2) and *I* ^2^ = 0% *p* = 0.012] (Figure 3).

## 4 Discussion

This study directly compared short‐term outcomes between two commonly utilized non‐crosslinked porcine acellular dermal matrices (PADMs), XenMatrix and Strattice, for open complex abdominal wall reconstruction. Our study found that XenMatrix mesh was associated with over 12 times the odds of developing an SSO postrepair compared to Strattice mesh. To our knowledge, this is the first study comparing head‐to‐head outcomes in patients who underwent VHR with XenMatrix or Strattice mesh.

Several studies have independently looked at outcomes of specific PADMs following VHR [12, 13, 32, 33, 34, 35, 36, 37]. Most of these studies have utilized PADMs to repair different grades of VHR, ranging from clean to contaminated. The reported rates of SSOs for XenMatrix has ranged between 0% in a study Byrnes et al. [13] and up to 21% in another study Pomahac et al. [12]. Although the rates of SSO for XenMatrix in our randomized and observational cohorts are higher than reported by these studies, there are key differences. For example, more than 75% of their patient cohort belonged to lower CDC wound classes, whereas on the other hand, in our study, the majority of our patient's cohort belonged to higher CDC classes. Similarly, two major studies have reported outcomes of Strattice mesh for VHR. The RICH trial reported 21% rates for both seroma and surgical site infection in their patient cohort [32]. Patel et al. reported 24.4% wound complication rates in their patient cohort [33]. These are consistent with the SSO rate in our observational cohort. Notably, our randomized cohort had lower than reported SSO rate. The combined (PADM) rate of SSO in our study was 26% and 40% for randomized cohorts and observational cohorts, respectively. This is consistent with what has been reported by previous studies [22, 38, 39]. Sivaraj et al. found the risk of SSO to be as high as 52.9% for porcine acellular dermal matrices (PADMs) [38].

Selecting a mesh with the best outcomes at the time of initial repair is critical in the reduction of increased healthcare costs associated with reoperation [5]. In terms of head‐to‐head comparison following multivariate analysis in the randomized cohort, Strattice appeared to be superior in reducing the risk of 6‐week SSO. Our meta‐analysis of these two findings favors Strattice mesh in the overall reduction of 6‐week risk of SSO, with no difference in the risk of hernia recurrence over the duration of study.

The rates of 30‐day reintervention and readmissions for SSO in our study were not statistically different for both meshes, these findings are in line with has been previously reported in the literature for biologic meshes [26]. Although synthetic meshes are currently preferred over biologic meshes due to several studies that have reported similar or even better outcomes with synthetic meshes over biologic meshes, even in contaminated fields [20, 26]. This preference is amplified by the cost of biologic meshes with a 30‐day direct hospital cost ∼$45,000, almost three times that of synthetic meshes (∼$17,000) [26]. However, this tradeoff comes off at a price. Biologic meshes remain excellent in terms of long‐term tissue integration, remodeling, and vascularization as well as cases where soft‐tissue augmentation is required [40]. This makes a valid argument for the continuous relevance of biologic meshes in complex abdominal wall reconstruction.

From a patient‐centered perspective, mesh choice must also consider health‐related quality of life (HRQoL) and long‐term economic impact. Multiple studies show that successful repairs, regardless of whether biologic, synthetic, or long‐term resorbable mesh is used, leads to large long‐term improvements in hernia‐specific and global HRQoL [41]. In the PRICE trial QoL substudy, biologic and synthetic mesh had similar improvements in patient‐reported outcomes over 2 years, but biologic mesh incurred significantly higher overall costs, with mean total episode‐of‐care costs of roughly $80,000 versus $61,000 for synthetic mesh [41]. Emerging long‐acting resorbable/biosynthetic meshes may offer an intermediate option, combining properties of synthetic meshes with excellent tissue integration, and promising HRQoL and complication profiles at a lower cost than biologics, but head‐to‐head comparative data remain limited, and follow‐up is still relatively short [15].

Importantly, this study emphasizes the importance of comparison of different brands of meshes within the same subgroup of biologics (i.e., xenografts). Historically, it is assumed that mesh with similar physical properties can be used interchangeably [42]; however, our study offers a different insight. The observed differences in outcomes between XenMatrix and Strattice, both non‐crosslinked porcine acellular dermal matrices (PADMs), may stem from variations in manufacturing processes [43, 44]. Experimental work has shown that relatively small changes in porcine dermal processing steps (detergents, enzymatic treatments, and sterilization methods) can significantly affect residual DNA content, ultrastructure, mechanical strength, and cytocompatibility of the resulting scaffold [43, 44]. More recent reviews on decellularized extracellular matrix and dermal matrices further emphasize that incomplete antigen removal and process‐induced matrix damage can modulate inflammatory signaling in vivo, with product‐specific differences in collagen fibril architecture and intrinsic mechanical properties potentially translating into different clinical performance [45, 46]. In this context, it is plausible that two superficially similar porcine ADMs may have intrinsic differences which result in different SSO profiles.

Our study has several limitations. First, due to the inability to complete patient enrollment into the RCT, and the small number of patients in the observational cohorts, our findings may be underpowered. Although *I* ^2^ values from the meta‐analysis were excellent, combining outcomes from both randomized and observational cohorts in the meta‐analysis may have introduced some level of bias and heterogeneity; therefore, the effect estimate of the RCT cohort alone should also be considered when interpreting results. Further, the imbalance in the number of patients randomized to both arms of the RCT occurred purely by random chance as appropriate randomization methods and adequate allocation concealment were employed. Although randomization ideally results in well‐balanced groups, minor imbalances can occasionally occur even with proper methods, particularly in studies with modest sample sizes. In this study, baseline characteristics between the two groups were generally comparable, and statistical analyses included appropriate adjustments for observed differences. However, multivariable analysis with low event counts have potential for overfitting and multivariable analysis results should be interpreted with caution. We believe these limitations had minimal impact on the validity of our findings. Finally, the recurrence rate may be underreported because not all patients underwent radiologic imaging at every follow‐up visit to objectively assess for recurrence.

## 5 Conclusions

Strattice meshes appears to be superior in the reduction of 6‐week risk of SSO following VHR compared to XenMatrix. Given that early SSO contributes to overall increase in healthcare burden, this may be helpful in optimal mesh selection for patients. Finally, larger randomized controlled trials of head‐to‐head studies are recommended to provide high level of evidence for the outcomes of VHR using biologic meshes.

## Author Contributions

**Abdulaziz Elemosho:** conceptualization, investigation, methodology, data curation, formal analysis, writing – review and editing, writing – original draft. **Benjamin A. Sarac:** conceptualization, methodology, data curation, investigation, formal analysis, writing – original draft, writing – review and editing. **Molly A. Olson:** formal analysis, writing – review and editing. **Ibrahim Khansa:** conceptualization, methodology, data curation, investigation, formal analysis, writing – original draft, writing – review and editing. **Paige N. Hackenberger:** conceptualization, methodology, data curation, investigation, formal analysis. **Vimal Narula:** conceptualization, methodology, data curation, investigation, formal analysis. **Daniel Eiferman:** conceptualization, methodology, data curation, investigation, formal analysis. **Jeffrey E. Janis:** conceptualization, methodology, data curation, investigation, formal analysis, project supervision, writing – original draft, writing – review and editing.

## Funding

The authors have nothing to report.

## Conflicts of Interest

The authors declare no conflicts of interest.

## Supporting information


Supporting Information S1

## Data Availability

Research data are not shared.
