# Supplementary material for: Comparative Outcomes of Two Non‐Crosslinked Porcine Acellular Dermal Matrices in Complex Abdominal Wall Reconstruction: A Randomized Controlled Trial and an Observational Cohort Study
Source: World J Surg. 2026 Feb 20;50(4):848–59. doi: 10.1002/wjs.70271 (PMC13070450; doi:10.1002/wjs.70271)
Supplement: Supplementary file 1 — Supporting Information S1 [file WJS-50-848-s001.docx]

**CONSORT Checklist**

| **Section / Item** | **Checklist Item** | **Where reported / Notes** |
| --- | --- | --- |
| **Title and abstract** |  |  |
| 1a | Identification as a randomized trial in the title | Title: “Comparative Outcomes of Two Non-crosslinked Porcine Acellular Dermal Matrices in Complex Abdominal Wall Reconstruction: A Randomized Controlled Trial and an Observational Cohort Study” |
| 1b | Structured summary of trial design, methods, results, and conclusions | Abstract: Background, Methods, Results, Conclusion. Trial design (randomized), interventions, primary/secondary outcomes, main results, and registration number clearly reported. |
| **Introduction** |  |  |
| 2a | Scientific background and explanation of rationale | Introduction (all paragraphs). Adequate background on biologic vs synthetic/bioresorbable mesh and rationale for head-to-head comparison of XenMatrix® vs Strattice®. |
| 2b | Specific objectives or hypotheses | Last paragraph of Introduction: objective to compare outcomes of XenMatrix® and Strattice® in VHR. |
| **Methods – Trial design** |  |  |
| 3a | Description of trial design (e.g., parallel), including allocation ratio | Methods – Study Design: randomized controlled trial in patients undergoing open elective complex abdominal wall reconstruction; allocation ratio 1:1 reported in “Sample Size, Randomization, and Blinding.” Trial is effectively a parallel-group design. |
| 3b | Important changes to methods after trial commencement, with reasons | Methods – Termination of Trial: early termination of RCT and addition of retrospective observational cohort with rationale (declining enrollment, formulary/financial issues). |
| **Methods – Participants** |  |  |
| 4a | Eligibility criteria for participants | Methods – Participants and Study Settings: adults ≥18 years, VHWG ≥2, elective repair, consent; exclusion criteria detailed with reference to full criteria in Supplementary Material. |
| 4b | Settings and locations where data were collected | Methods – Participants and Study Settings: patients recruited and surgical procedures performed at The Ohio State University Wexner Medical Center. |
| **Methods – Interventions** |  |  |
| 5 | The interventions for each group with sufficient details to allow replication | Methods – Surgical Intervention: operations performed by senior author with general surgeon co-author(s), standardized perioperative protocol, component separation, preference for primary closure, drainage in all patients; mesh type clearly defined (XenMatrix® vs Strattice®). |
| **Methods – Outcomes** |  |  |
| 6a | Completely defined pre-specified primary and secondary outcome measures, including how and when they were assessed | Methods – Outcome Measures & Data Collection: primary outcome = 6-week SSO; secondary outcome = hernia recurrence ≥1 year follow-up; fully defined components of SSO (skin/fat necrosis, wound dehiscence, infection, hematoma, seroma, enterocutaneous fistula); recurrence definition; follow-up schedule (weekly for one month, then every three months to one year). |
| 6b | Any changes to trial outcomes after the trial commenced, with reasons | No changes to primary or secondary outcomes after trial commencement. |
| **Methods – Sample size** |  |  |
| 7a | How sample size was determined | Methods – Sample Size, Randomization, and Blinding: power analysis using prior XenMatrix® and Strattice® studies, α=0.05, power 80%, SD=3, target n=70 (35 per arm). |
| 7b | When applicable, explanation of any interim analyses and stopping guidelines | Early stopping explained in Termination of Trial; No planned interim analyses and formal stopping rules. |
| **Methods – Randomization: sequence generation** |  |  |
| 8a | Method used to generate the random allocation sequence | Methods – Sample Size, Randomization, and Blinding: computerised random number generator in Microsoft Excel. |
| 8b | Type of randomisation; details of any restriction (e.g., blocking, stratification) | Simple randomization without blocking or stratification was used |
| **Methods – Randomization: allocation concealment mechanism** |  |  |
| 9 | Mechanism used to implement the random allocation sequence (e.g., sealed envelopes), describing any steps taken to conceal the sequence until interventions were assigned | Methods – Sample Size, Randomization, and Blinding: sequentially numbered, sealed, opaque envelopes opened after confirming eligibility and consent. |
| **Methods – Randomization: implementation** |  |  |
| 10 | Who generated the random allocation sequence, who enrolled participants, and who assigned participants to interventions | Methods – Sample Size, Randomization, and Blinding: allocation sequence generated a priori by team member not involved in recruitment or intraoperative decision-making; patients enrolled and randomized after eligibility and consent confirmed. (Enrolment vs assignment roles are implied but not separately labelled.) |
| **Methods – Blinding** |  |  |
| 11a | If done, who was blinded after assignment to interventions and how | Methods – Sample Size, Randomization, and Blinding: single-blinded study; patients not informed which mesh they received; surgeons and OR staff were aware. Postoperative clinical assessments and data abstraction were performed by treating surgeons and research staff who were not blinded to the assigned mesh, whereas patients remained blinded throughout follow-up. |
| 11b | If relevant, description of the similarity of interventions | The two meshes were handled and implanted using the same operative principles and perioperative pathways, which helped maintain patient blinding |
| **Methods – Statistical methods** |  |  |
| 12a | Statistical methods used to compare groups for primary and secondary outcomes | Methods – Statistical Analysis: logistic regression (simple and multivariable) for 6-week SSO; adjustment variables pre-specified; random-effects meta-analysis; Kruskal–Wallis and chi-square tests for baseline comparisons. |
| 12b | Methods for additional analyses, such as subgroup analyses and adjusted analyses | Methods – Statistical Analysis: covariate-adjusted models and combined random-effects meta-analysis clearly described; no subgroup or interaction analyses reported. |
| **Results – Participant flow** |  |  |
| 13a | For each group, the numbers of participants who were randomly assigned, received intended treatment, and were analysed for the primary outcome (a flow diagram is strongly recommended) | Results – “A total of 66 patients, 46 from RCT, and 20 from retrospective review, were identified and included in analysis.” Group-specific numbers for XenMatrix® vs Strattice® are provided for demographics and outcomes. Figure 1. |
| 13b | For each group, losses and exclusions after randomisation, together with reasons | No patients were excluded from the analyses for protocol violations, however three patients from XenMatrix group were excluded (one from the randomized trial, and two from the observational cohort) due to an incomplete 6-week outcome data. |
| **Results – Recruitment** |  |  |
| 14a | Dates defining the periods of recruitment and follow-up | Methods – Study Design: February 2015 to August 2022; follow-up periods by group reported in Results (means, SDs, ranges). |
| 14b | Why the trial ended or was stopped | Methods – Termination of Trial: explains early termination due to declining enrollment and institutional issues around biologic mesh formularies. |
| **Results – Baseline data** |  |  |
| 15 | A table showing baseline demographic and clinical characteristics for each group | Results – Patient Demographics and Baseline Characteristics; references to Table 1a (RCT) and Table 1b (observational). |
| **Results – Numbers analysed** |  |  |
| 16 | For each group, number of participants (denominator) included in each analysis and whether analysis was by “intention-to-treat” or per-protocol | Results: total analysed given for RCT and observational cohorts, and group sizes for XenMatrix® vs Strattice®. All randomized patients who received mesh and had available 6-week follow-up were analysed according to their randomized assignment, and all observational patients who met eligibility criteria were analysed according to the mesh they received. The primary analyses used a complete-case approach; no outcome data were imputed |
| **Results – Outcomes and estimation** |  |  |
| 17a | For each primary and secondary outcome, results for each group and the estimated effect size and its precision (e.g., 95% CI) | Results – Surgical Outcomes (RCT and observational) and Meta-Analysis: 6-week SSO proportions, adjusted ORs with 95% CIs and p-values for each cohort and pooled; recurrence proportions reported. |
| 17b | For binary outcomes, presentation of both absolute and relative effect sizes | Relative effect sizes (ORs and CIs) are provided for SSO, with group percentages |
| **Results – Ancillary analyses** |  |  |
| 18 | Results of any other analyses performed, including subgroup analyses and adjusted analyses, distinguishing pre-specified from exploratory | Results – Meta-Analysis: covariate-adjusted combined analysis reported; no subgroup or post-hoc exploratory analyses. |
| **Results – Harms** |  |  |
| 19 | All important harms or unintended effects in each group | Results – Surgical Outcomes: SSO components, reinterventions, and readmissions reported by group. |
| **Discussion** |  |  |
| 20 | Trial limitations, addressing sources of potential bias, imprecision, and, if relevant, multiplicity of analyses | Discussion – Limitations: incomplete RCT enrollment, small observational cohort, potential bias from combining RCT and observational data, chance imbalance, possible under-reporting of recurrence. |
| 21 | Generalisability (external validity, applicability) of the trial findings | Discussion: comments on relevance of mesh choice, biologic vs synthetic/bioresorbable meshes, and brand-specific differences suggest implications for broader practice; explicit statement on single-centre nature and generalisability is implicit but not separately labelled. |
| 22 | Interpretation consistent with results, balancing benefits and harms, and considering other relevant evidence | Discussion and Conclusions: interpretation of higher SSO with XenMatrix®, no difference in recurrence, and contextualization with literature on biologic, synthetic, and resorbable meshes; acknowledges limitations and avoids over-statement. |
| **Other information** |  |  |
| 23 | Registration number and name of trial registry | Abstract and Methods – Participants and Study Settings: ClinicalTrials.gov NCT02228889. |
| 24 | Where the full trial protocol can be accessed, if available | **Trial protocol is available in** ClinicalTrials.gov NCT02228889 as well as on request. |
| 25 | Sources of funding and role of funders | This research did not receive any specific grant |

***Inclusion Criteria***

All surgical candidates were optimized according to a previously described protocol.^21^ All participants provided voluntary informed consent prior to enrollment, in accordance with IRB-approved study protocols. Eligible patients were adults over 18 years old, suitable for elective hernia repair with VHWG grade 2 or above, good performance status without active life-threatening cardiac disease, pulmonary disease, renal disease, or hematologic disease. Patients needing resection of large abdominal wall tumors using biologic mesh for closure were also included.

***Exclusion criteria:***

Patients who did not provide consent were excluded from the study. Other criteria for exclusion were patients with known allergy to porcine products, active nicotine users (within the past 4 weeks) presenting for elective hernia repair, patients with active life-threatening cardiac disease, pulmonary disease, renal disease, hematologic disease presenting for elective hernia repair, patients presenting for emergent hernia repair (in the setting of bowel strangulation, necrosis, penetrating trauma) as it would be difficult to consent those patients for the study preoperatively, and patients with severe systemic sepsis.

*Datapoints Collected*

Data points collected include patient demographics (age, gender, follow-up data, body mass index BMI, and comorbidities like diabetes, hypertension and COPD), patient baseline characteristics, (primary hernia diagnosis, previous hernia repair (with or without mesh) and number of previous hernia repairs, hernia dimension), operative data (total OR time and ASA class, type of hernia diagnosis, Center for Disease Control (CDC) wound class, Ventral Hernia Working Group (VHWG) grade, Kanters grade23, primary fascial closure, components separation, and mesh position).
